# Highly automated driving: the role of visuo-attentional and executive abilities in take-over success

**DOI:** 10.3389/fpsyg.2025.1685223

**Published:** 2025-12-01

**Authors:** Damien Schnebelen, Franck Mars, Camilo Charron, Sami Mecheri, Régis Lobjois

**Affiliations:** 1 COSYS-PICS-L, Université Gustave Eiffel, Marne-la-Vallée, France; 2 Nantes Université, Centrale Nantes, CNRS, LS2N UMR CNRS 6004, Nantes, France; 3 Université de Rennes 2, Rennes, France; 4 Département Neurosciences et Sciences Cognitives, Institut de Recherche Biomédicale des Armées, Brétigny-sur-Orge, France

**Keywords:** take-over, executive abilities, visuo-attentional abilities, highly automated driving, individual differences

## Abstract

Previous studies have sought to better understand exogenous factors explaining drivers’ ability to regain control or not from highly automated driving. However, few studies have examined the role of individual factors in drivers’ take-over behavior and performance. The present study sought to examine the extent to which take-over performance can be predicted by individual differences in visuo-attentional and executive abilities. After a period of automated driving on a simulator, participants aged 20 to 60 (*N* = 118) had to regain control of their vehicle in a critical take-over situation (i.e., avoiding an obstacle by changing lanes while other vehicles are approaching at higher speeds). The take-over manoeuvre was considered successful if the participants managed to complete it without colliding with the obstacle or another vehicle. All participants completed a battery of cognitive (working memory, inhibitory control, attentional flexibility, and planning) and visuo-attentional tests (visuomanual coordination, multiple object avoidance, and multiple object tracking), and reported their age and driving experience. Several partial least-squares models predicting the success of the manoeuvre from individual abilities were compared. The most accurate model had an accuracy of 70.79% and identified spatial working memory (measured with the CORSI task), visuomanual coordination (one’s ability to manually track a moving target) and driving experience (annual mileage) as key factors for the success of the take-over. Conversely, higher inhibitory control ability, as measured by the Flanker and Stop-Signal tasks, was negatively related to take-over success, possibly because these participants exerted strong cognitive control over the non-driving task during automated driving, which came at the cost of flexibility.

## Highlights

- The likelihood of collision during take-over was examined through individual abilities.
- A multi-level assessment of executive and visuo-attentional abilities was conducted.
- Working memory appeared to be the key determinant of take-over success, followed by visuomanual coordination and driving experience.
- On the other hand, greater inhibitory control predicted a higher risk of collision.

## 1 Introduction

The progressive automation of vehicles has introduced a series of challenges in terms of human–machine cooperation, ranging from situations where the driver fully assumes the role of operator without assistance (Level 0 according to the Society of Automotive Engineers; SAE International, 2016) to situations where a fully automated system assumes total control of the driving task (SAE Level 5). This cooperation becomes particularly critical in the context of highly automated driving (SAE Level 3), where the driver is still expected to resume control upon request when the automated system encounters a situation that exceeds its operational design domain (e.g., the sudden appearance of an obstacle on the roadway). The transition period during which control is transferred from the automated system back to the human driver is referred as the take-over process.

Take-over from highly automated driving has received extensive research, with a primary focus on the impact of exogenous variables, i.e., those directly related to the take-over situation (Mole et al., 2019). These factors can be grouped into three main categories: (1) the driving situation at the moment of the take-over request—such as the presence of an incident (Merat et al., 2012), traffic density (Gold et al., 2016), or road geometry (So et al., 2021); (2) the human–machine cooperation modalities during the take-over—such as the time budget available for the driver to regain control (Gold et al., 2013; Vlakveld et al., 2018) or the sensory modalities of the take-over alert (Petermeijer et al., 2017); and (3) the driver’s state at the time of the take-over request—such as engagement in a secondary task (Zeeb et al., 2016), time spent in automated driving (Bourrelly et al., 2019), or repeated exposure to take-over requests (Samani et al., 2022). Meta-analyses have provided clarification regarding the impact of these factors on various dimensions of take-over performance. Concerning take-over times (e.g., the time taken by the driver to achieve effective vehicle control or to direct their gaze toward the road following the alert), Zhang et al. (2019) demonstrated that drivers regain control more rapidly when facing an emergency situation, when they are not using a handheld device or engaged in a visually demanding secondary task, when they have prior experience with take-over manoeuvres, and when the take-over alert is visual. Regarding both take-over time and some indicators of take-over quality (e.g., the occurrence of a collision or vehicle stability), Gold et al. (2018) found that performance is enhanced when drivers are afforded ample time to react, when traffic is absent during the take-over, and when drivers have prior take-over training.

Still, during this critical phase, the driver is required to re-engage both motor and cognitive resources in the driving task before resuming vehicle control (for models of re-engagement, see Zeeb et al., 2015; Deniel and Navarro, 2023). Endogenous factors, i.e., individual-specific characteristics, can therefore have a significant influence on take-over performance. Among these factors, age, gender and driving experience were primarily examined. Driving experience is the only factor that has produced consistent findings: experienced drivers—whether in terms of overall driving practice or familiarity with advanced driver assistance systems—consistently demonstrated superior take-over quality compared to novices (Chen et al., 2021; Zeeb et al., 2015, 2016; Zhang et al., 2023). However, take-over times were similar between novices and experienced drivers (Chen et al., 2021; Zhang et al., 2023). In contrast, the influence of age was more mitigated (Gasne et al., 2022). The meta-analyses from Zhang et al. (2019) and Gold et al. (2018) discussed earlier concluded that age explains only a small portion of the variance in take-over performance. While some studies have reported faster reaction times among younger drivers (Li et al., 2018, 2019), such differences were not observed (Clark and Feng, 2017; Körber et al., 2016) or only partially replicated (Lee and Kang, 2023). Findings regarding gender remained also inconclusive. Samani et al. (2022) observed that male drivers exhibited longer take-over times compared to female drivers, whereas Lee and Kang (2023) found this effect only in response to an auditory take-over alert. Furthermore, So et al. (2021) suggested that gender effect may hold for younger drivers but tends to reverse among older drivers. Some studies even pointed to the opposite direction: Chen et al. (2024) showed a tangential effect of gender, with females having longer take-over times than males.

Although these biographical factors provide relevant insights, they do not directly capture the motor, cognitive, and visual processes underlying the takeover. Zeeb et al. (2016) investigated how the difficulty of a secondary, non-driving-related task influenced both takeover time and quality. They found that, while the time required to return hands to the steering wheel was unaffected by the secondary task, takeover quality was significantly impaired. This suggests that takeover motor response time is little affected by distraction, contrary to the preceding cognitive processing underlying the transition and determining takeover quality. Connected to this, Sahaï et al. (2021) examined the effects of different autonomous driving training programs—written materials, tutorials, and driving practice—on takeover performance and showed that participants who underwent practical training regained control of the vehicle faster than those in other groups. However, practical training did not produce any positive effect on drivers’ visual behavior likely to facilitate takeover quality (no measure of takeover quality *per se* was included in the study). The authors argued that training primarily improves takeover response time at the latest motor stages, rather than enhancing perceptual or cognitive readiness. Echoing the results of Zeeb et al. (2016), this suggests that the main challenge of the takeover lies in the perceptual and cognitive demands rather than in the response during intervention execution (see also Vlakveld et al., 2018).

Visuo-cognitive abilities — encompassing visual perception, attention, and decision-making — are thus crucial in the context of automated driving, where rapid and accurate information processing is essential for safely resuming control. Consequently, any delay caused by task switching, distractions, or divided attention can significantly compromise a driver’s ability to react within the critical hazard response window. A clear understanding of these key functions and their role is therefore essential for designing safer, more intuitive human-machine interfaces and for minimizing the risks associated with takeover.

Schnebelen et al. (n.d.) (submitted) addressed this issue by analysing the brain regions activated during a non-critical take-over, compared to a fully automated driving condition without a take-over request. Using fMRI, the authors found that, during take-over, drivers exhibit increased activation in brain areas involved in visual information processing (occipital cortex), motor preparation and execution (supplementary motor area), as well as regions associated with visuomotor coordination (cerebellum). Regaining control of the vehicle also engaged the prefrontal cortex, which plays a central role in executive functions—such as working memory, inhibition, and cognitive flexibility (Friedman and Robbins, 2022; Funahashi and Andreau, 2013)—as well as in decision-making processes (Euston et al., 2012; Manes et al., 2002). Schnebelen et al. (n.d.) (submitted) also compared brain areas recruited immediately after the transition compared to a baseline drive in manual driving. The main differences between the two conditions relied on visuomotor and visual areas. The authors suggested that immediately after recovering control, visuomotor coordination is not as efficient as it is after prolonged manual driving. This aligns with Mole et al. (2019), who stated that one key factor of take-over success is the ability to recover efficient visuomotor coordination and recalibrate perceptual-motor control.

In their study, Peng et al. (2022) investigated the specific role of cognitive processes involved during take-over by assessing participants’ abilities prior to the driving task, focusing on executive functions—inhibition, working memory, and cognitive flexibility. These abilities were then examined in relation to age and take-over performance in scenarios where drivers were either engaged in a secondary task or not. The results revealed that, when drivers were engaged in a secondary task, vehicle lateral stability was positively associated with cognitive flexibility. Conversely, in the absence of a secondary task, take-over performance improved with working memory capacity.

Although this approach is novel to highly automated driving, predicting road user’s behavior (drivers, pedestrians) based on individual differences in cognitive and perceptual-cognitive tests is an active area of research. Numerous studies have demonstrated that reduced performance on a visual test such as the Useful Field of View (UFOV) is a significant predictor of risky driving behavior, whether among young (e.g., McManus et al., 2015) or older drivers (e.g., Wood et al., 2012). More recently, attention has turned toward the Multiple Object Tracking (MOT) paradigm as a means to evaluate attentional abilities relevant to driving given its closer resemblance to the demands of real-world driving than UFOV (Alberti et al., 2014; Mackenzie and Harris, 2017; Michaels et al., 2017). The MOT task requires individuals to follow several moving targets simultaneously among distractors, engaging multiple aspects of attention—including selective, sustained, and divided attention—within dynamic visual environments (Vater et al., 2021). Empirical studies have shown that, in moderate workload driving scenarios, lower MOT performance has been linked to increased crash risk, greater variability in lateral positioning, and reduced driving speeds (Michaels et al., 2017). Conversely, individuals with higher MOT scores tend to make fewer driving errors (Mackenzie and Harris, 2017). In addition to MOT, the Multiple Object Avoidance (MOA) task has also been proposed as a potential predictor of driving behavior, adding a visuomotor component by requiring participants to control an object while avoiding collisions with others. Results indicated that MOA scores correlated significantly with driving performance. MOA scores were also associated with specific oculomotor patterns—such as broader horizontal visual scanning and faster, more pronounced saccades—which reflect more effective attentional strategies in complex driving situations (Mackenzie and Harris, 2017). Together, these findings underscore the relevance of MOT and MOA tasks as tools for assessing attentional resources critical to safe driving. They not only help predict driving performance but also offer insight into individual differences in how attentional capacity supports adaptive responses under increasing task demands.

Overall, previous research examining the role of endogenous factors in take-over performance has primarily focused on individual biographical characteristics such as age, gender, and driving experience. However, few studies have investigated the underlying processes involved in take-over in order to assess their impact on performance. In this field, it has been shown that motor control is not in itself a major issue, unlike the re-engagement of perceptual and cognitive processes. The present study was therefore designed to contribute to this area by going beyond previous work on several levels. First, to our knowledge, only one study (Peng et al., 2022) has explored the relationship between executive functions and take-over performance. One objective of this research was thus to provide new evidence regarding the role of executive functions in participants’ ability to successfully take over control of an autonomous vehicle. Furthermore, recent work has shown that visuo-attentional abilities—measured using dynamic tests involving multiple dimensions of attention—are positively correlated with driving behavior (e.g., Mackenzie and Harris, 2017). A second objective was therefore to investigate the extent to which visuo-attentional abilities contribute to successful take-over performance. More broadly, the study aimed to explore the combined role of a set of endogenous factors by integrating biographical, cognitive, and visuo-attentional variables.

To this end, participants aged between 20 and 60 completed a battery of six cognitive tests targeting various components of executive functioning, and three visuo-attentional tests covering different dimensions of visual attention. The autonomous driving and take-over task were performed in a driving simulator using a particularly demanding scenario in terms of attention and decision-making (Marti et al., 2022). The contribution of all variables to the success in performing the takeover without colliding with other vehicles was evaluated by comparing multiple partial least-square regression models. In addition, an a posteriori assessment of drivers’ situational awareness at the moment of take-over was conducted as a complementary measure to aid in interpreting the results.

## 2 Materials and methods

### 2.1 Participants

One hundred and eighteen participants were recruited for this study. They were aged between 20 and 60 years old, had a valid driving licence for at least two years, drove regularly (measured by the number of km travelled during the last two years, with a minimum of 2000 km) and had normal or corrected-to-normal vision. Five participants were excluded from the final analysis due to either technical problems or improper take-over manoeuvres (e.g., swerving to emergency lane, loss of vehicle control, no response to take-over request). Therefore, 113 participants (44 females) were considered in the data analysis.

All participants were informed that they were free to withdraw at any time, for any reason. They gave written informed consent and were unaware of the hypotheses under investigation. The study was approved by the Ethics Committee of Université Gustave Eiffel. The principles of the Declaration of Helsinki and the French regulation for data protection (RGPD) with regard to experimentation were respected.

### 2.2 General procedure

Participants were first welcomed in the laboratory and gave written informed consent to be involved in the study. They also reported their age and driving experience. The entire study lasted approximately two hours and consisted in three parts, which were counterbalanced across participants.

One part consisted of driving on a simulator, during which participants drove a highly automated vehicle and experienced a sudden take-over request, as described and illustrated hereafter (30 min approximately). Another part was dedicated to assess participants’ visuo-attentional abilities (35 min approximately). During the third part of the experiment, participants performed a battery of cognitive tests measuring executive functions (55 min approximately, with one break halfway through). To avoid fatigue effects, these three parts were separated by long breaks.

### 2.3 Driving task

#### 2.3.1 Driving simulation

**Figure 1:**
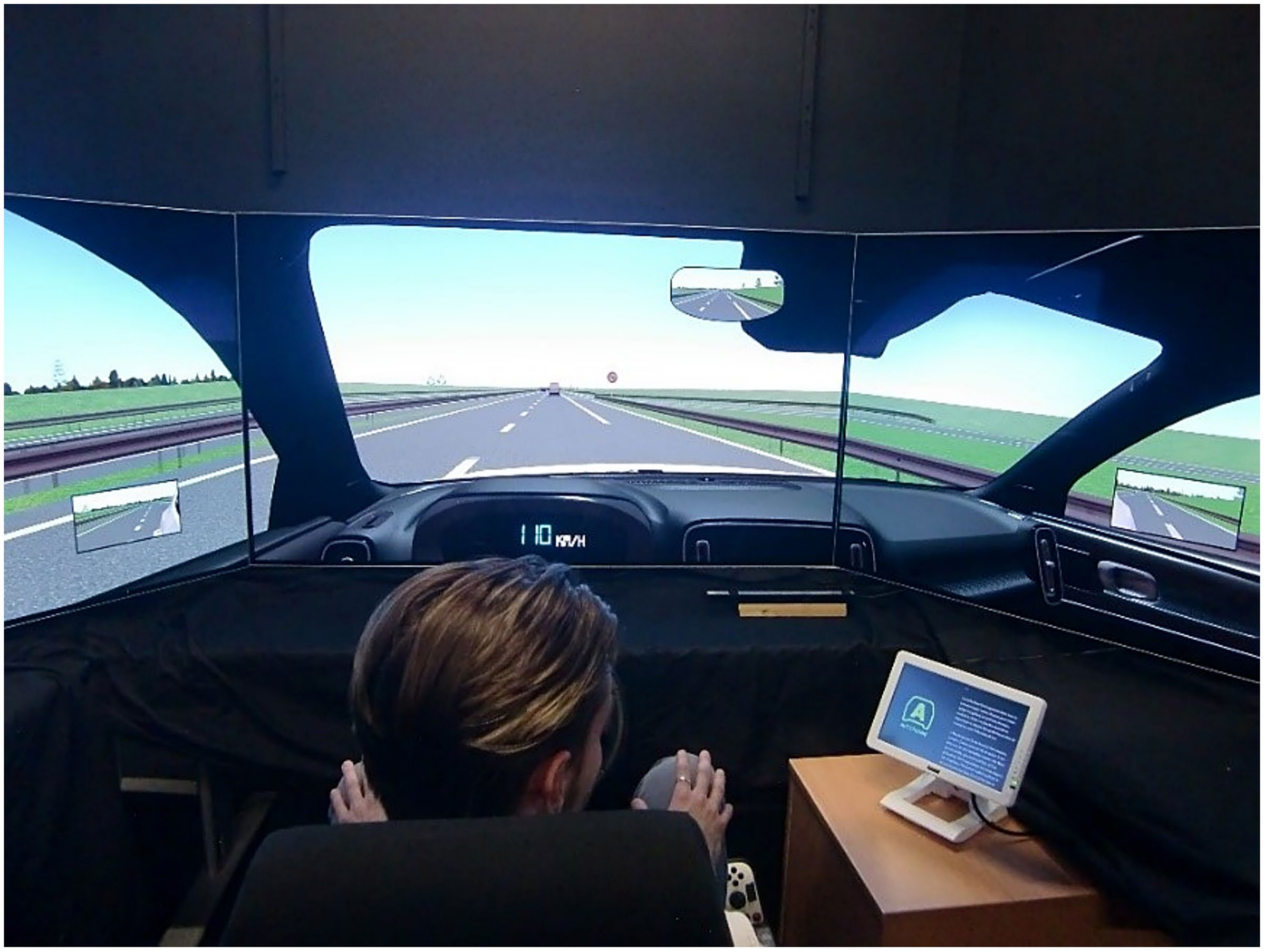
Driving simulator setup.

The simulator (Figure 1) consisted of a steering wheel with force feedback, a set of pedals and a gear lever developed by Logitech (model G25) and an adjustable seat. An audio feedback system (not spatialised) provided auditory information on engine speed. Three-dimensional renderings and driving scenarios were generated using custom software developed in Unity. The driving scene was displayed on three 55-inch screens (4 K resolution) covering 140° of the participants’ horizontal field of vision. An additional small screen was located on the right side of the participant. A camera also recorded the entire driving scene during the experiment.

#### 2.3.2 Automation and HMI

During the experiment, a level 3 automated vehicle was simulated. The vehicle had an automation feature that could be activated by the driver when prompted by the system. Then, the vehicle regulated its own speed and position in the lane, managed overtaking by other road users and complied with regulations. However, when the vehicle encountered a situation that it could not manage, a take-over request was sent to the driver. This request consisted of a visual alert on the side screen and an audio prompt from the vehicle. Drivers then had eight seconds to regain control by pressing a pedal, turning the steering wheel, or pushing a button on it. Once they had regained control of the vehicle, they remained in charge until they activated autonomous driving again.

#### 2.3.3 Instructions and driving scenarios

Once the participants had settled into the driving simulator, they were explained level 3 automation and the ways in which they could interact with the vehicle, as described above. They were also informed that, during automated driving, they might perform a secondary task, consisting in reading a text displayed on the side screen. Finally, they were informed that they would experience two driving scenarios: one for training purposes and one for the experiment itself.

In the training scenario, participants drove manually for eight minutes on a suburban and on a motorway section. On the motorway section, participants tested twice the activation and deactivation of the autonomous mode in non-critical situations. This training step allowed participants to get used to the virtual environment, to the controls of the simulator and to the take-over procedure. It was also useful to adjust the scrolling speed of the text on the side screen, so that reading was fluid but demanding for each participant.

The experimental scenario was adapted from the study by Marti et al. (2022), which reported a take-over situation that split the participants in two relatively equivalent group based on their take-over performance (59% of participants collided another vehicle, 41% succeeded without any collision).

**Figure 2:**
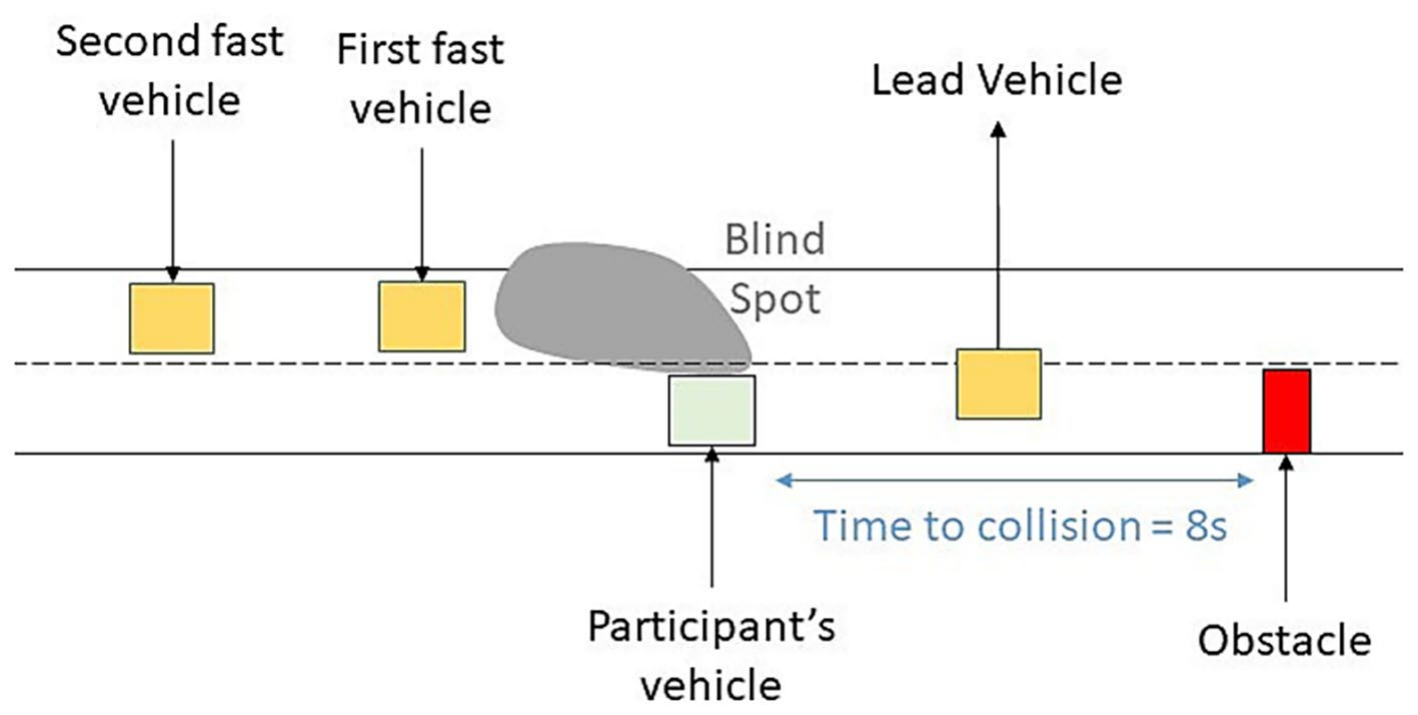
Illustration of the driving situation at the moment of the take-over request.

The scenario itself took place on a dual carriageway. After one minute in manual driving, participants had to activate the autonomous mode. One minute after activation, they started reading a text on the side screen. From this moment, the participant’s vehicle followed a lead vehicle at a fixed speed of 110 km/h in the right-hand lane. The reading time lasted approximately 6 min and stopped automatically 2 s before the take-over request. The driving situation at the moment of the take-over request is presented in Figure 2.

A take-over was requested due to the presence of an obstacle (i.e., a stationary vehicle blocking the lane). The obstacle had been masked by the lead vehicle prior to the take-over request was made, and was revealed when the lead vehicle moved to the left lane, triggering the take-over request. From that moment, the participant had an eight-second time headway before reaching the obstacle. Two other vehicles, approaching in the left-hand lane at a higher speed (120 km/h) than the participant, were also involved in the take-over scenario. At the moment of the take-over request, the two fast vehicles were visible in the rear-view mirror. However, the first fast vehicle was in the blind spot three seconds after take-over request. Assessing its location and speed was therefore only possible between the end of the reading task (2 s prior to take-over request) and before its presence in the blind spot (3 s after take-over request). To successfully regain control, the driver had three solutions: 1. Adjust their speed and swerve to the left lane between the two fast vehicles, 2. To reduce speed further and change lane after the second fast vehicle, or 3. To brake hard and bring the vehicle to a stop before colliding with the obstacle.

#### 2.3.4 Take-over quality assessment

There is a wide range of indicators of take-over quality in the literature (for a review, see Mole et al., 2019). Some of them focus solely on the participant’s vehicle (e.g., steering stability), while others report on the vehicle’s situation with respect to events or other vehicles during take-over (e.g., time-to-collision between the participant’s vehicle and an obstacle). In the present study, take-over quality was represented by a binary vector *Success*. If the participant did not collide with any vehicle, it took the value 1. In the event of a collision with any vehicle (obstacle, approaching vehicles), it took the value 0. This indicator was particularly relevant in view of the take-over scenario. First, it may represent the integrated time-to-collision value between the participant’s vehicle and the other three vehicles (two fast vehicles and one stationary obstacle). It could also be directly linked to driver safety during the take-over.

#### 2.3.5 Situation awareness score

**Figure 3:**
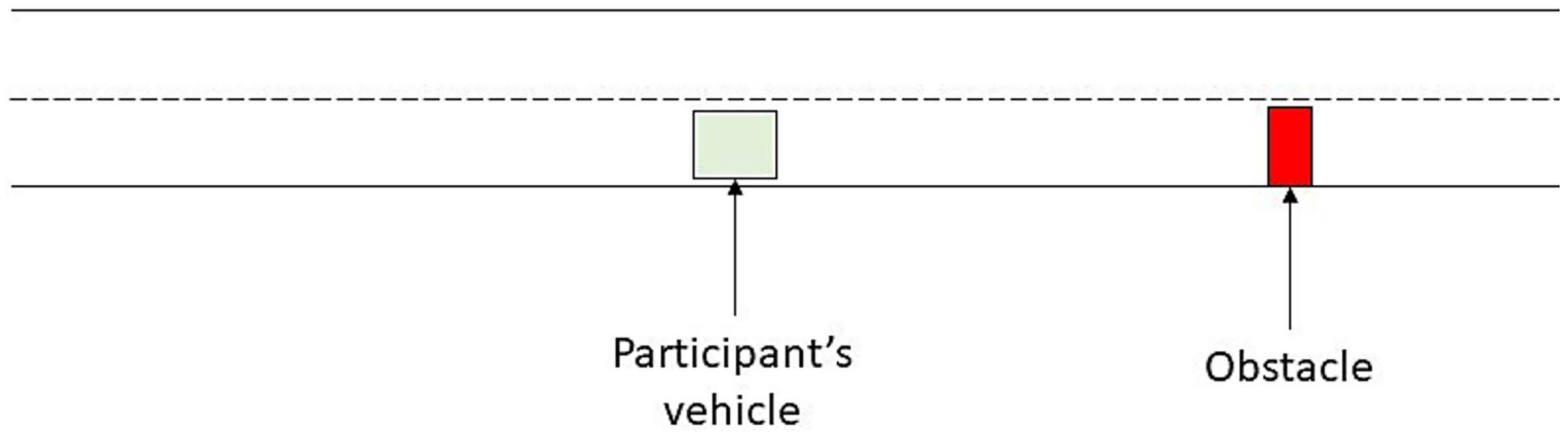
Partial representation of the driving situation for situation awareness assessment.

Immediately after driving, a partial representation of the driving situation at the take-over request was presented to the participants. This representation only included the location of the obstacle and the participant’s vehicle (see Figure 3).

The participants were instructed to indicate other vehicles they had seen at the moment of the take-over request, as well as their speed relative to their own vehicle (higher, equal or lower). Participants could add as many vehicles as they wanted, although only three vehicles were expected: the two fast vehicles approaching on the left-hand lane and the lead vehicle. A situation awareness (SA) score, ranging from 0 (no vehicle placed correctly or with incorrect speeds) to 3 (all vehicle placed correctly with the correct speeds), was then calculated. This score was introduced to assess the driver’s mental state at the moment of take-over but does not constitute an individual characteristic. Therefore, it was not included as a predictor of take-over success in the modelling work described below.

### 2.4 Visuo-attentional tests

The aim of these tests was to evaluate participant’s visuo-attentional abilities using three complementary tasks targeting different components of visual attention. First, the *Visuomanual Coordination (VMC)* task assessed sensorimotor coordination and the ability to maintain control over a moving target, thus evaluating basic sensorimotor ability. In contrast, the *Multiple Object Avoidance (MOA)* task assessed dynamic visual attention and spatial monitoring, as participants had to continuously distribute their gaze and attention across the visual field to avoid collisions with multiple moving objects. Finally, the *Multiple Object Tracking (MOT)* task engaged several levels of visual attention (selective, sustained and divided; Vater et al., 2021) as well as working memory by asking the participants to track the motion of multiple objects simultaneously. Altogether, these tasks offered a multi-level assessment of visuo-attentional processes, ranging from simple visuomotor tracking to complex attentional deployment, as summarized in Table 1. An illustration and description of each task is provided in Supplementary Figure 1.

**Table 1:** Visuo-attentional processes involved in each task.

	Visuomanual coordination task (VMC)	Multiple object avoidance task (MOA)	Multiple object tracking task (MOT)
Visual tracking	X	X	X
Visuomanual coordination	X	X	
Divided visual attention		X	X
Working memory			X

Prior studies showed that these tasks are able to capture inter-individual differences on task performance in ecological contexts requiring visual attention with high spatio-temporal constraints, like sports or driving. In sports, a higher performance at VMC was found for experienced ball players (Mallek, 2019; Mallek et al., 2017). Similarly, Mangine et al. (2014) showed a correlation between MOT scores and performances of professional basket-ball players. In the driving context, performance at the MOA was shown to predict more effective visual scanning strategies of the road and safer driving behavior (Mackenzie et al., 2022; Mackenzie and Harris, 2017). Furthermore, MOT performance was shown to be a good predictor of individual differences in driving behavior (Mackenzie and Harris, 2017; Michaels et al., 2017).

All the tasks were performed on a computer and displayed on a 24-inch screen. Before data recording, the instructions for each test were explained to the participants, who then underwent training for the task. During the training step, participants could adjust the sensitivity of the mouse (if necessary).

#### 2.4.1 Visuomanual coordination

In this task, participants had to track a ball following linear or curved trajectories and bouncing off the edges of a rectangle with the mouse cursor. Each trial started with a red ball in the centre of the screen. When participants overlaid the mouse cursor to the ball, it started to move in an unpredictable direction for 12 s. In half of the trials, bouncing the ball off the edge altered its trajectory, making the task more challenging for the participant. In all cases, the participant had to keep the mouse cursor as close to the ball as possible at all time. For the test itself, participants performed 12 trials. Performance in the task corresponded to the percentage of time spent close to the ball (mouse-ball distance ≤ 0.5 cm).

#### 2.4.2 Multiple object avoidance

The MOA task consisted of participants controlling a sphere using a mouse so as to avoid collisions with other spheres (of different colour) moving on linear trajectories, bouncing off the edges of the workspace and potentially colliding with each other.

Each trial started with 3 spheres to avoid. After an initial period of 15 s, spheres were added one by one and the period of time for each new stage was extended (20 s with four spheres, 30 s with five spheres, etc.) making the task increasingly difficult. The participant’s aim was to avoid colliding with one of the other spheres for as long as possible. Participants completed a total of six trials. The performance metric for this task corresponded to the time elapsed before a collision occurred (average total time over the six trials).

#### 2.4.3 Multiple object tracking

The MOT task involved participants visually tracking four targets among four distractors inside a 3D space (cube). The task was carried out using NeuroTracker™ (CogniSens) run in 3D mode, with participants wearing anaglyph 3D glasses. At the beginning of each trial, all balls were stationary and the targets were highlighted for 2 s. Then, objects moved linearly in the 3D space for 8 s. At the end of a trial, the balls remained stationary, and participants had to select the four targets initially presented. Feedback was then given to the participants.

Participants completed the CORE assessment, which consisted of three blocks of twenty trials. The speed of the objects increased or decreased in subsequent trials depending on whether participants identified all target objects correctly in the previous trial. The outcome measure was a visual tracking speed threshold that was automatically calculated by the NeuroTracker software. This speed threshold was used to measure participant’s performance in the task.

### 2.5 Cognitive tests

The study from Peng et al. (2022) evaluated participants’ executive functions using three tests. Working memory was assessed with the N-back task, inhibition abilities were measured with the Simon task, and the Task switching ability tested with a homemade task-switching paradigm. This approach, while relevant, does not consider the diversity of mechanisms involved in executive functions. For example, the inhibitory control may be investigated either in terms of visual inhibition (e.g., relating participant’s performance to focus on relevant information despite visual distractions) or response inhibition (e.g., assessing participant’s ability to cancel a pre-planned action, when necessary, like the Simon task). Similarly, working memory can likewise be evaluated by examining participant’s ability to update and monitor visual information (e.g., N-back task), auditory information (e.g., auditory N-back task), or spatial information (e.g., Corsi task).

The present study included these nuances in a battery of six cognitive tests extracted from the literature. Two of them assessed participant’s inhibitory control ability. The first one (Flanker Task; Eriksen and Eriksen, 1974) focused on visual inhibition whereas the second one (Stop-Signal Task; Verbruggen et al., 2019) focused on response inhibition. Similarly, two tests were set up to investigate visual working memory (N-back; Kirchner, 1958) and spatial working memory (Corsi task; Fischer, 2001). Cognitive flexibility was measured by the Trail Making Test (TMT; Sánchez-Cubillo et al., 2009). Finally, the Tower of London task assessed spatial planning ability (Shallice, 1982). An illustration and description of each task is provided in Supplementary Figure 2.

In the end, these tasks captured a rich set of executive processes (inhibitory control, working memory, cognitive flexibility, and spatial planning) offering a multi-level assessment of an individual’s higher-order cognitive functions. The tasks were implemented in Psychopy (Peirce, 2007) or jsPsych (de Leeuw, 2015) or directly on paper boards. Before data recording, the instructions for each task were explained to the participants, who then underwent training for the task.

#### 2.5.1 Inhibition

##### 2.5.1.1 Flanker task

Flanker Task measured participants’ ability to inhibit distractors (Eriksen and Eriksen, 1974). On each trial, five arrows appeared simultaneously, and the participant had to indicate as quickly as possible the orientation (left, right) of the central arrow by pressing the corresponding arrow keys on a keyboard. The trials could be congruent, i.e., the central arrow was pointing in the same direction as the others, or incongruent in the opposite case. As part of the study, participants completed two blocks of 40 successive trials. Performance corresponded to the percentage of successful trials and the difference in response time between congruent and incongruent trials.

##### 2.5.1.2 Stop-signal task (SST)

Stop-Signal Task measured participants’ response inhibition ability. The task consisted of an alternating sequence of standard trials (75% of the time) and stop trials (25% of the time). For a standard trial, an arrow (pointing to the left or right) appeared in white in the centre of the screen. As soon as the subject saw the arrow, they had to indicate its direction by pressing the appropriate arrow touch on the keyboard as quickly as possible. In the stop trials, the arrow appeared white at first but suddenly turned red after a variable amount of time. In that case, the participants had to inhibit their motor response. The stop signal delay (i.e., the time of appearance of the red arrow) was adaptive between blocks, targeting a success rate of 50% at stop trials for each participant. Participants carried out 64 trials divided into 4 blocks. Performance in this task corresponded to the stop signal reaction time (SSRT), computed with the integration method as recommended by Verbruggen et al. (2019). As SSRT reflects the speed of inhibitory process, a higher value indicated lower abilities.

#### 2.5.2 Working memory

##### 2.5.2.1 N-back task

The N-back Task was used to assess participants’ visual working memory. A sequence of coloured squares scrolling across the screen was presented to the participants. They had to compare the square they saw with the one they had seen N time before, and pressed a key on the keyboard if the two squares were identical. Three levels of difficulty were used here: 1-back, 2-back and 3-back. Performance in the task corresponded to the percentage of correct answers and the difference in response times between the 3-back (the most difficult) and the 1-back (the simplest) conditions, the latter serving as an individual calibration.

##### 2.5.2.2 Corsi task

Corsi Task focused on participants’ spatial working memory (Fischer, 2001). The participants’ task was to remember, and then reproduce, a precise sequence of squares appearing up in white on a grid of 25 squares displayed on the screen. The task had a variable level of difficulty: if the participants reproduced the sequence correctly, it was made more complex on the next trial by adding a square. If not, the task was repeated with another sequence of the same difficulty. The task was repeated as long as the participants did not fail twice in a row at the same level of difficulty. Performance in this task corresponded to the final level reached by the participant, i.e., to the number of spatial items participants were able to remember and replace in the forward order.

#### 2.5.3 Cognitive flexibility

The aim of the Trail Making Test was to assess participants’ ability to switch between tasks (Sánchez-Cubillo et al., 2009). Participants had to connect dots on paper boards as quickly as possible and without error in two consecutive steps. For the calibration step, dots contained numbers to be connected from the smallest to the largest. For the test step, dots contain numbers and letters to be connected by alternating numbers and letters from the smallest to the largest (e.g., 1-A–2-B, etc.). As the number of errors was very low, only the time difference between the test board (TMT-B) and the calibration board (TMT-A) was kept for data analysis.

#### 2.5.4 Spatial planning

Tower of London was used to assess participants’ spatial planning ability. The task consisted of reproducing a model by moving balls on a virtual board. The board contained 3 balls (red, green and blue) stacked on cylinders. The cylinder furthest to the left could hold a maximum of 3 balls, the one in the middle 2 balls and the one on the right a single ball. The participant had to manipulate the balls one by one with the mouse and reproduce the model in as few actions as possible in a limited time (20s). The participant therefore had to study the model, plan the actions to be taken and carry them out within this timeframe. Twelve different models were presented, ranging from the simplest (minimum of 3 ball movements) to the most difficult (minimum of 8 ball movements). The data analysed for this task were the number of models correctly reproduced by the participant and, for these successful models, the number of additional ball movements (compared with the minimum number of movements).

### 2.6 Data analysis

#### 2.6.1 Summary of the data

Take-over performance was represented by a binary vector *Success,* which takes the value 0 if the participant’s vehicle collided with another vehicle, and 1 otherwise.

Fifteen variables measuring the individual abilities were used for data analyses (summarised in Table 2). For tests measuring tasks performance and response time (N-back, Flanker, Tower of London), two variables of performance were considered, instead of one variable for all other tests. A matrix abilities, containing 113 rows (one per participant) and 15 columns (one per variable) gathered the data on participants’ abilities. In the case of a negative relationship between the variable and task performance, the numerical data was converted to negative values. With this modification, a higher value for each variable corresponds to a higher ability. The table showing the correlations among all test scores, as well as between each score and takeover performance, is presented in Supplementary Table 1.

**Table 2:** List of all the variables in the matrix abilities.

Data type	Abilities	Task	Variable	Unit
Biographical data	Experience	—	Age	year
	Driving experience	—	Number of kilometers travelled per year	1,000 km/year (inclusion minimum: 2)
	Driving experience	—	Number of years since passing the driving licence	Year (inclusion minimum: 2)
Visuo-attentional abilities	Visuomanual coordination	Visuomanual coordination	Percent of time spent close to the ball across the trials	Percent
	Visuomotor attention	Multiple Object Avoidance	Mean time calculated on all trials	Second
	Visuo-cognitive attention	Multiple Object Tracking	Visual tracking speed	a.u
Executive abilities	Working memory	N-Back	Difference in accuracy between 3-Back and 1-back	Percent
	Working memory	N-Back	Difference in mean time between 3-Back and 1-back	ms
	Working memory	Corsi	Highest level reached at the task	n.a
	Inhibition	Stop-Signal Task	Stop-Signal Reaction Time	ms
	Inhibition	Flanker	Percentage of successful trials	Percent
	Inhibition	Flanker	Difference in mean time between congruent and incongruent trials	ms
	Cognitive Flexibility	Trail Making Test	Time difference between dual-task and simple task	s
	Spatial planning	Tower of London	Number of successful trials	n.a
	Spatial planning	Tower of London	Number of unnecessary moves	n.a

To examine which individual abilities—assessed through executive and visuo-attentionnal tests as well as biographical questionnaires—contribute to take-over performance, and to what extent, two complementary approaches were conducted. The first approach consisted of performing t-tests comparing participants’ test battery scores (i.e., measures of individual abilities) as a function of their take-over success. These univariate analyses were designed as exploratory assessments, aimed at identifying potential associations between take-over success and performance on each test considered individually, prior to the multivariate modelling stage. The second approach sought to predict take-over success from the entire matrix of individual ability measures using a multivariate modelling framework based on multiple Partial Least Squares (PLS) logistic regressions. All analyses were performed using R (R Core Team, 2018).

#### 2.6.2 First approach

The first approach consisted in performing between-group comparisons for each ability of the matrix abilities. The idea was to investigate whether each metric taken individually allows distinguishing between the groups of participants who collided (*Success* = 0) and those who did not (*Success* = 1). To that purpose, independent t-tests were conducted. No statistical correction for multiple testing was applied since the purpose of this analysis was to explore associations between individual abilities and take-over performance.

To investigate whether participants in the two groups exhibited similar level of situation awareness, a complementary analysis based on an independent t-test was also performed on the situation awareness score. However, situation awareness score was not included in the modelling approach described below, as it is context-dependent, whereas the other predictors were measured independently of driving performance.

#### 2.6.3 Modelling approach

The modelling approach was based on multiple model comparisons of prediction models. To predict *Success* from *Abilities*, we performed PLS Logistic Regression (package plsRglm). This analysis consisted of the following three main steps.

##### 2.6.3.1 Finding the optimal structure of data prediction

The first step of PLS regression was to find an optimal structure (based on components) that supports data prediction. A large number of components provides a robust prediction, with few errors, but it is specific to the dataset: if the dataset slightly changes, the prediction power may fall drastically. On the other hand, a low number of components provides a lower prediction but is less likely to be affected by small variations on the datasets. The optimal structure is the one that offers the best balance between the two.

To compute the optimal number of components, we compared prediction models with different structures (from one to fifteen components). The performance of the models was assessed using the maximum number of false positive (FP: the model predicted a success, but the participant failed at the critical case) or false negative (FN: the model predicted a failure, but the participant successfully managed the critical scenario) with a leave-one-out procedure. In the end, the optimal number of components was selected from the model minimizing the maximum number of FP and FN (Minimax method; Minimax = min (max (FP; FN))). In case of equality between two models, the one with the better prediction (i.e., the one minimizing the number of errors) was selected. This optimal number of components was used in all subsequent steps of data analysis.

##### 2.6.3.2 Model selection

The second step aimed at identifying the most relevant abilities to predict *Success*. To do so, a set of 2^15–1 models were compared, with each model having as input matrix a partial set of the matrix abilities. For instance, the first model tried to predict *Success* from 15 abilities (the complete data set), the 15 following models tried to predict Success from 14 abilities, etc., until the last 15 models predicted *Success* from only one ability. In this step as well, model performance under leave-one-out validation was evaluated based on the maximum number of FP and FN, and model comparisons were conducted according to the Minimax criteria.

##### 2.6.3.3 Analysis of the final model

After model selection, the final prediction of *Success* from the partial set of Abilities was performed on the data from all participants. PLS logistic regression coefficients were interpreted as follows: a positive coefficient indicated that this ability tended to increase the prediction value. Inversely, a negative coefficient tended to reduce the prediction value. Given the nature of the regression (logistic), the weight of one ability for the prediction was given by the exponential value of its coefficient. This provided a multiplier of the probability of Success when the ability increases: if performance on one test increased by one unit while performance on the other abilities remained similar, the probability of success was multiplied by this value.

## 3 Results

Sixty-three participants did not collide with any vehicles in the take-over scenario, and constituted the Success group. The majority of successful participants (41 out of 63) either stopped or greatly reduced their speed before swerving into the left lane after the second fast-approaching vehicle. The remaining 22 participants managed to merge between the two vehicles without incident. On the other hand, fifty participants collided with at least one vehicle, mainly with the first fast vehicle. Theses participants constituted the Fail group.

### 3.1 First approach

**Figure 4:**
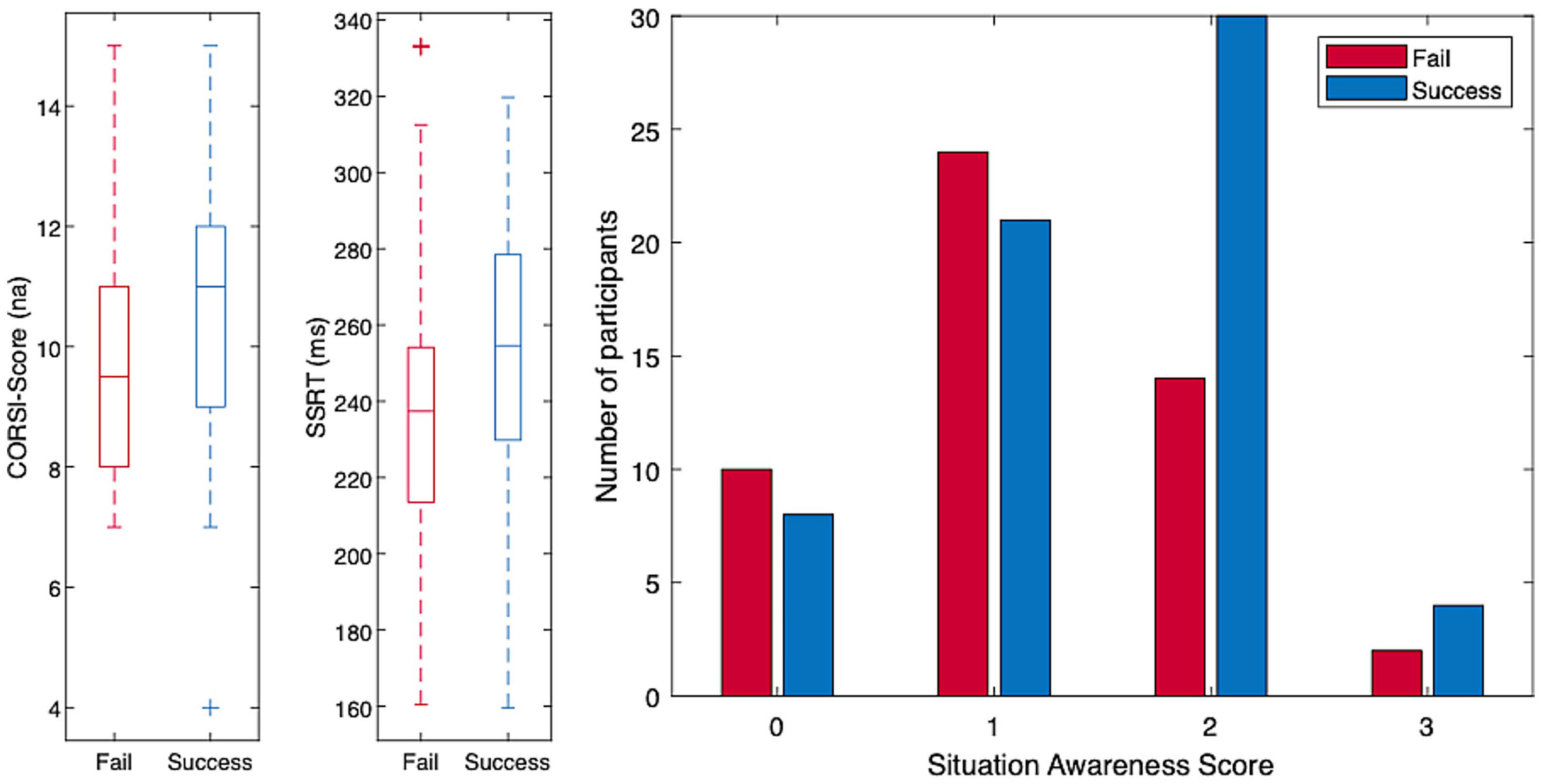
Left: box plot of the two indicators providing significant differences (corsi-score, SSRT) between the groups of participants (fail, success). Right: distribution of the situation awareness scores as a function of participants’ performance at take-over (fail; success).

Only two significant group differences were observed among the visuo-attentional and executive measures. Participants in the Success group achieved higher scores on the Corsi task and exhibited longer Stop-Signal Reaction Times (SSRT) compared to those in the Fail group (t(111) = 2.25, *p* = 0.03 and t(111) = 2.11, *p* = 0.04, respectively). It should be noted, however, that these differences would no longer reach significance if corrections for multiple comparisons were applied. The boxplots for those indicators are presented in Figure 4 (left). Participants in the Success group also achieved higher situation awareness scores (see Figure 4, right), compared to the participants in the Fail group (t(111) = 2.10, *p* = 0.04). A complete picture of the box plots for all indicators as well as the full table of t-tests can be found in Supplementary Figure 3 and Supplementary Table 2, respectively.

### 3.2 Modelling approach

#### 3.2.1 Construction and selection of the optimal model

##### 3.2.1.1 Construction of the optimal structure (choice of the number of components)

Table 3 summarizes the results of model comparisons to determine the optimal structure of the model (in terms of number of components). According to the minimax criteria, models with one or two components are equivalent. However, the total number of errors was smaller with one component. Thus, one component was considered as the optimal model structure.

**Table 3:** Analysis of the performances of prediction (maximum number of errors; total number of errors) of the model as a function of the structure of the model (number of components) and the number of predictors.

Number of components of the model	1	2	3	4	5 to 15
Number of predictors	15	15	15	15	15
Maximum number of errors (false positive or false negative)	28	28	29	31	30
Total number of errors	45	48	54	55	55

##### 3.2.1.2 Model selection

Given the large number of possible models (2^15^–1), detailed results are provided in Supplementary Figure 4.

According to the Minimax criteria, two models were retained. Still, the comparison between the models (see Table 4) yielded to the selection of Model B for the final step, referred hereafter as the optimal model. It is worthy to note here that the two models were based on close predictors: they have a common set of five predictors, but Model A included one additional variable: the visual tracking speed (MOT performance).

**Table 4:** Comparison of the two best models of prediction.

Models	Model A	Model B (selected)
Number of components of the model (obtained at the previous step)	1	1
Number of predictors	6	5
Maximum number of errors (false positive or false negative)	21	21
Total number of errors	36	33

An additional permutation analysis was conducted to assess whether the relationships identified by the PLS model was robust. Specifically, the input dataset was modified by randomly shuffling all predictor variables (i.e., the variables corresponding to our five indicators) and applying them to the model 10,000 times. For each iteration, we recorded the number of classification errors and compared the performance of these permuted-data models to that of the model built on the original, unshuffled data. In the vast majority of cases, models based on permuted data exhibited approximately 10 additional errors compared to the original model. When examining the distribution of these differences, we found that only 1% of the permuted datasets resulted in fewer than 4 additional errors, and—most importantly—none of the 10,000 permutations achieved a performance equal to or better than the model using the original data (i.e., every permuted model made at least one additional error). These results indicate that the performance of our PLS model cannot be explained by random associations among predictors.

#### 3.2.2 Analysis of the optimal model

The confusion matrix of the optimal model is given in Table 5. The prediction accuracy was 70.79%.

**Table 5:** Confusion matrix of the prediction.

		Predicted success (1)	Predicted fail (0)
Take-over performance	Success (1)	50	13 (FN)
	Fail (0)	20 (FP)	30

The optimal model was based on five indicators, given in Table 6. The results suggest that three abilities—Visuospatial Working Memory, Visuomanual Coordination and Driving Experience (annual mileage)—increased the probability of Success, whereas one ability (Inhibition) reduced it.

**Table 6:** Abilities relevant for success prediction.

Abilities	Task	Variable	Unit (1 SD)	Coefficients	Interpretation in terms of probability of success
Driving experience	—	Number of kilometers per year	17,584 km/year	0.22	24%
Visuomanual coordination	Visuomanual coordination	Percent of time spent close to the ball across the trials	8.87%	0.35	42%
Working memory	Corsi	Highest level reached at the task	2	0.45	56%
Inhibition	Stop-signal task	Stop-signal reaction time	39.3 ms	−0.42	−34%
Inhibition	Flanker	Percentage of successful trials	2.79%	−0.36	−30%

## 4 Discussion

This study examined the relationship between drivers’ individual abilities and their success in a critical take-over situation. These abilities were assessed using standardized tests commonly employed in the literature, covering a large spectrum of visuomotor, attentional and executive processes. The take-over scenario required drivers to regain control of their vehicle following engagement in a secondary task (reading task). A take-over was considered successful if the participant avoided colliding with both a stationary obstacle in their lane and vehicles overtaking in the left-hand lane.

In the first analytical step, we examined whether each individual test could, on its own, distinguish between successful and failed take-over manoeuvres. The results revealed very few significant effects—and none that remained significant after correcting for multiple comparisons. This pattern of largely non-significant univariate results motivated the use of a PLS modelling approach, which simultaneously considers all predictors and can uncover multivariate patterns that may not be detectable through individual tests.

In the second analytical step, a combination of individual capacities that best predicted success in the critical take-over scenario was identified by comparing multiple logistic PLS regression models. The final model, selected for its predictive performance, retained five key indicators: driving experience (annual mileage), working memory capacity (final level achieved in the Corsi task), visuomanual coordination performance (percentage of time spent accurately tracking a moving target), and inhibitory control (stop-signal reaction time and percentage of correct responses in the Flanker task). This model achieved a predictive accuracy of 70.8%.

### 4.1 Take-over performance predictors

#### 4.1.1 Executive functions and take-over performance

The results suggest that visuo spatial working memory (Corsi task) significantly contributed to the prediction of take-over success. In contrast, inhibition ability (SSRT and Flanker task) has a negative impact on take-over success. Cognitive flexibility and spatial planning did not emerge as relevant predictors.

Working memory, which reflects an individual’s capacity to maintain and process information simultaneously, was identified as a key factor increasing the likelihood of successful take-over performance. This finding is consistent with Peng et al. (2022), who found that when drivers were not engaged in a secondary task at the moment of the take-over request, those with higher working memory capacity exhibited greater lateral stability during take-over. Similar results were reported by Mäntylä et al. (2009) in young drivers, showing that those with stronger working memory abilities demonstrated better lane-keeping performance. Peng et al. (2022) suggested that participants with low working memory capacity struggle to process dynamic visual information (e.g., speed and lane position), which in turns reduced their stability on the road. In the context of our take-over situation, it is most likely that working memory capacity supported the driver’s ability to rapidly acquire relevant situational information and develop situation awareness. Indeed, participants had only five seconds to build this awareness, including the first fast vehicle (2 s between the end of the secondary task and three seconds after the take-over request). This hypothesis is further supported by the complementary analysis of situation awareness scores: drivers who successfully regained control without a collision exhibited higher SA scores. However, it is important to emphasize that SA should not be considered a direct predictor of performance, nor a cognitive state determined by the same underlying factors as performance itself. Rather, it provides a complementary perspective that helps interpret the behavioral outcomes observed in this study.

On the other hand, the model highlighted the critical role of inhibitory control in predicting failure in the critical take-over. Participants who scored higher in the Flanker task, which measured the ability to suppress distracting information, and the Stop-Signal task, which assessed response inhibition, were more likely to have a collision. This negative association contrasts with the findings of Peng et al. (2022), who did not report any link between inhibitory control and take-over performance. There may be three possible reasons for this difference. First, Peng et al. (2022) has employed a Simon Task, which examines the effects of interference during stimulus–response mapping conflicts. Stop signal task, on the other hand, examines how well a response which has already been initiated can be cancelled. Thus, task paradigms look at different aspects of response inhibitory control: reactive inhibition (response stopping) for the SST, and interference inhibition during stimulus–response incompatibility for the Simon Task. Second, the secondary task in the study by Peng et al. (2022) was chosen as auditory (0-back task) in order “to avoid additional visual distraction in the traffic environment.” Drivers thus looked toward the road environment during autonomous driving, and did not have conflicting visual information to inhibit. This may have reduced the need to suppress information coming from the road environment, leading them to exert less cognitive control on the secondary task since there was little need to protect it against interference. In our study, in contrast, participants had to read a dynamic text that required them to direct visual attention outside of the road environment, and to protect it from other dynamic visual information coming from the road environment. Arguably this led drivers to more strongly impose the secondary task-set (more cognitive stability, see Egner, 2023) to inhibit driving-related visual information. Third, in further support of this, drivers in Peng et al. (2022) performed several take-overs, influencing their switch readiness in order to adapt to more changing demands (higher take-over likelihood) at the expense of cognitive stability. In fact, an increase in cognitive flexibility is synonymous with a decrease in cognitive stability (e.g., Dreisbach, 2012; Dreisbach and Fröber, 2019; Goschke, 2003, 2013; Hommel, 2015; Paul et al., 2021). For example, switch costs increase on trials following incongruent stimuli—requiring stability (Goschke, 2000; Meiran, 1996; Monsell et al., 2003; Rogers and Monsell, 1995). Switch costs are also reduced when switches are more frequent in a block of trials (e.g., Kang and Chiu, 2021; Liu and Yeung, 2020; Siqi-Liu et al., 2022; Siqi-Liu and Egner, 2020), showing that encountering a context implying more switch leads to increased switch readiness. These findings show that individuals adapt their cognitive stability to changing demands, enhancing stability when interference from distractors is higher (here, dynamic visual information coming from the road environment while executing a visual task with dynamic stimuli) and when task switches are less probable (here, unique rather than multiple take-over requests).

In the context of our critical take-over situation, such stability could prove detrimental. First, it may hinder their ability to disengage from the mental set associated with the secondary task. Practically, these participants might continue fixating on the secondary task display even after completing the reading task, resulting in a delayed visual reorientation to the driving scene. This behavior was observed in the study by Marti et al. (2022), in which some drivers persisted in looking at the secondary task screen even after its completion. This hypothesis could be further explored using eye-tracking measures. Second, even when the gaze is finally redirected on the driving scene, they might miss key visual information. Indeed, Jia et al. (2024) demonstrated that during the take-over process, drivers primarily focus their visual attention on the road ahead, often neglecting peripheral visual information from side and rear-view mirrors. This tendency may be amplified in drivers with strong inhibitory control, as they are more likely to inhibit peripheral inputs perceived as non-relevant and distractors. However, in that kind of situation, peripheral vision offers critical elements for successful take-over performance, such as the presence of nearby vehicles on adjacent lanes.

Contrary to the findings of Peng et al. (2022), cognitive flexibility did not emerge as a relevant predictor of take-over success in the present study. This discrepancy may be explained by differences in the driver’s state at the moment of the take-over request. In Peng et al.'s (2022) study, drivers were actively engaged in a secondary task when the take-over alert was issued. In contrast, in our study, the secondary task ended two seconds before the alert, thereby reducing the time pressure associated with task switching. This reduced urgency could explain why cognitive flexibility indicators did not contribute to predicting performance in the present take-over scenario. Additionally, this divergence might also come from the nature of the assessment tool used: the cognitive flexibility test (TMT) employed in our study is relatively simple and may not fully capture the cognitive demands associated with a complex take-over situation. A custom task-switching paradigm, for example using the presentation of driving scenes as in the study of Schuch et al. (2020), may be particularly suited to that purpose. This is another avenue worth exploring in follow-up studies.

#### 4.1.2 Visuo-attentional abilities and take-over performance

In manual driving, visuomanual coordination is essential: drivers visually track specific points on the road ahead (for a unified approach, see Lappi and Mole, 2018) to control vehicle and maintain appropriate lateral positioning. During take-over situations, as the driver is out of the loop (Merat et al., 2019), Mole et al. (2019) hypothesized that recovering effective visuomotor coordination was a key determinant of successful vehicle control resumption. In line with this, previous studies have shown that passive drivers — i.e., individuals observing the driving scene without exerting control over the vehicle — exhibit different visual sampling strategies compared to active drivers in manual driving (Mackenzie and Harris, 2015; Mars and Navarro, 2012; Navarro et al., 2016). The findings from our study are in line with this hypothesis, as visuomanual coordination abilities are among the factors that predict take-over success.

On the other hand, more complex visuo-attentional abilities (visuomotor attention, visuo-cognitive attention) did not significantly contribute to take-over performance. It is therefore plausible that performing good take-over mostly depends on low-level visuo-attentional processes. This hypothesis is consistent with the fMRI study of Schnebelen et al. (n.d.) (submitted), who found no high-level brain areas involved in either manual driving after take-over or in long-term manual driving.

It is also possible that the assessment of visuo-attentional abilities only partially reflects the reality of our take-over situation. Indeed, if the MOT test is engaging in terms of working memory, participants started with a full awareness of the situation (number of balls, location and speed) and try to keep that awareness along the trials. This would be consistent with their implications during manual driving, where the drivers update visual information continuously (Mackenzie et al., 2022; Mackenzie and Harris, 2017; Michaels et al., 2017). On the contrary, the take-over requires the driver to switch from a passive non-driving task to steering actively and to rapidly assess the driving situation, almost from scratch. With this perspective, complementary visuo-attentional tests focusing on the building of situation awareness from the drivers may be indicated.

#### 4.1.3 Driving experience and take-over performance

Driving experience has been extensively studied in the context of manual driving showing that experienced drivers generally have smoother trajectories and more efficient visual scanning strategies (Lehtonen et al., 2014; Navarro et al., 2023; Robbins and Chapman, 2019). In the context of automated driving, driving experience also contributes to the take-over quality (Chen et al., 2021; Zhang et al., 2023). Our results both support and nuance this finding: the only experience-related factor that significantly predicted success in our critical take-over scenario was the annual mileage (number of kilometres per year). In contrast, neither age nor driving licence seniority had a significant effect, despite their frequent correlation with experience.

Based on previous results, the contribution of driving experience to regaining control of an automated vehicle may be to shape driving style or to facilitate more efficient responses in high-demand contexts. Indeed, de Winter et al. (2024) and Matsumuro et al. (2020) have observed that manual driving style influences take-over performance in automation. McDonald et al. (2019) have also reported that drivers tend to execute take-over manoeuvres that closely resemble their responses to hazardous events in manual driving. On the other hand, driving experience may also contribute to situation awareness assessment. Indeed, Wright et al. (2016) drew a parallel between driving experience and situation awareness: experienced drivers are better at achieving situation awareness during take-over. Taken together, these results emphasise that driving experience likely contributes by influencing behavioral and visual patterns in hazardous situations like the take-over.

### 4.2 Model interpretation

#### 4.2.1 Components of the model

A previous model investigating the role of executive functions was presented by Peng et al. (2022). In this study, principal component analysis (PCA) was employed to identify components, yielding three distinct components for each domain of executive functions (inhibitory control, task switching, and working memory). Subsequently, driving performance—measured by steering variability—was correlated with the scores on each component, allowing the authors to underline the relative importance of individual executive functions.

The methodological approach adopted in the present study—PLS logistic regression—provides a more direct means of addressing the research question by simultaneously extracting latent components representing both individual capacities and performance (i.e., success in the critical take-over scenario). Given the diversity of predictive indicators, which covered a broad spectrum of executive and visuo-attentional abilities, it was plausible that multiple components would be required (e.g., a executive component and a visuo-attentional component). However, model comparisons indicated that the most accurate prediction of take-over success was achieved through a single component. The final model should therefore be interpreted like this: there is a unique underlying ability to take-over, integrating a visuo-attentional dimension (visuomanual coordination), an executive dimension (spatial working memory) and driving experience.

#### 4.2.2 Relative proportion of the predictors

Interestingly, only the Stop-Signal Reaction Time (SSRT) and the score at the Corsi task significantly distinguished participants who failed from those who managed in the critical take-over scenario. None of the other individual indicators, when considered separately, were able to differentiate between these groups. However, the approach employed to analyse the data demonstrated that a set of five indicators collectively predicted well take-over performance. While these five indicators should be considered as a whole, the coefficients obtained from the PLS regression allow for the interpretation and ranking of individual indicators (and thus the corresponding abilities) based on their relative importance in the prediction.

As a result, spatial working memory emerged as the primary factor contributing to successful take-over performance. The second key predictor was visuomanual coordination. Finally, driving experience played a more modest, tertiary role in predicting successful performance. Conversely, individuals’ inhibitory control abilities had a negative impact on take-over success.

#### 4.2.3 Model performance

The best predictive model of take-over success achieved a classification rate of 70.8%. This optimal model also showed that individual abilities were better at predicting take-over success (model specificity = 79.4%) than take-over failure (model sensitivity = 60.4%).

These results suggest on the one hand that individual abilities significantly contribute to take-over success, with a predictive performance approximately 20% above random level. This indicates that drivers are not equally prepared to handle a critical take-over situation based on their individual abilities.

On the other hand, individual abilities alone do not fully account for drivers’ performance during take-over. Especially, take-over failure is difficult to predict from individual abilities assessed outside of the driving simulator. Therefore, incorporating additional context-related endogenous variables could improve predictive accuracy. In this regard, drivers’ visual behavior moments before the take-over request might be particularly relevant, as drivers engaged in a visual secondary task are less likely to successfully regain control (Deniel and Navarro, 2023; Zhang et al., 2019). Furthermore, eye-tracking measures provide a more direct assessment of situation awareness, despite the potential limitations posed by the “look-but-fail-to-see” phenomenon. It may also be relevant to investigate how drivers physically perform the vehicle take-over (e.g., placing their hands on the steering wheel prior to effectively regaining control). Additionally, more general driving habits such as driving styles could offer valuable insights for the prediction.

However, incorporating such variables requires continuous data collection on driver behavior, making this approach relevant in experimental contexts but more challenging in on-road environments. Nonetheless, recent attempts have demonstrated the potential of this approach. For example, by leveraging convolutional neural networks (CNN), Liu et al. (2024) predicted take-over safety (safe/unsafe) using a pupillometry-based indicator, achieving a prediction accuracy of 81.98%. Similarly, Du et al. (2020) predicted take-over performance (good/bad) from visual and physiological data, the best model achieving a prediction accuracy of 84.3%.

### 4.3 Advantages and limitations

In prior literature, most take-over studies employed within-subject designs in which drivers perform several take-overs under experimentally controlled driving conditions. Although this approach allows a large amount of data to be collected with a relatively small sample of participants, it introduces the risk of overlearning effects. Indeed, prior research has demonstrated that repeated exposure to take-over scenarios significantly reduces the likelihood of collisions (Gold et al., 2018) and decreases drivers’ reaction times to take-over requests (Samani et al., 2022).

In contrast, the present study employed a single-trial design in which each driver performed only one critical take-over event (following a training phase), thereby creating more ecologically valid conditions. In real-world scenarios, critical take-overs are expected to be rare that may impact swich readiness and thus reinforce the difficulty to switch. To ensure a robust dataset, a large sample of participants (*N* = 113) was recruited, and their cognitive and visuo-attentional resources were assessed using standardized tests from the scientific literature.

The primary limitation of this study concerns the generalizability of its findings to other driving scenarios. While some result patterns align with those reported by Peng et al. (2022), notable differences remain. These divergences can likely be attributed to variations in the take-over scenarios, such as whether a secondary task was ongoing at the time of the take-over request, the driving context at the moment of the alert (e.g., the number of vehicles involved, driving environment), or the specific characteristics of the take-over event itself (e.g., time available between the alert and the obstacle, modality of the alert). In both studies, however, working memory played a key role in explaining take-over performance. Future work is needed to confirm the implications of other executive functions in take-over as a function of the driving situation.

## 5 Conclusion

This study investigated the relationship between drivers’ executive and visuo-attentional abilities and their performance in a take-over situation after a period of automation. Using PLS regression, multiple model comparisons revealed an optimal model predicting success from a set of abilities. Working memory emerged as the key determinant of take-over success, followed by visuomanual coordination and driving experience. Conversely, driver’s inhibition abilities were negatively related to the likelihood of collision during take-over. The optimal model’s predictive power was well above the random level, but incorporating other endogenous variables, such as gaze direction before the take-over request, could further improve performance. Future studies with take-overs in diverse driving context are required to confirm these findings.

## Data Availability

The raw data supporting the conclusions of this article will be made available by the authors, without undue reservation.
