# Supplementary material for: Highly automated driving: the role of visuo-attentional and executive abilities in take-over success
Source: Front Psychol. 2025 Dec 1;16:1685223. doi: 10.3389/fpsyg.2025.1685223 (PMC12702763; doi:10.3389/fpsyg.2025.1685223)
Supplement: Supplementary file 1 [file Table_1.docx]

| **VISUOMANUAL COORDINATION TASK – Visuomanual Coordination**  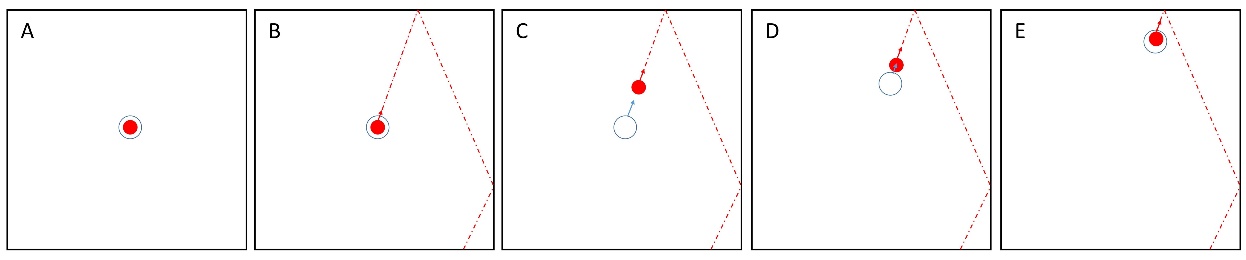  A) When the red ball appeared on the centre of the screen, participant had to place the mouse cursor on it. B) A random trajectory was attributed to the red ball. C, D and E) Participants had to track the ball as accurately as possible during twelve seconds with the mouse cursor. |
| --- |
| **MULTIPLE OBJECT AVOIDANCE (MOA) TASK – Visuomanual Attention**  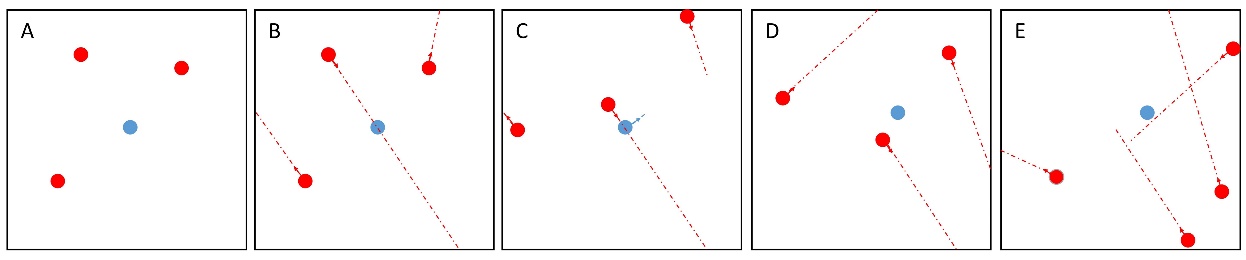  A) The trial started with 3 static red balls while the participant controlled the blue ball with the mouse. B) After few seconds, the red balls moved around with different trajectories but a similar speed. C) Participants had to avoid collision with a red ball by moving the mouse cursor. D) Collision was avoided. E) After a few seconds, a new red ball was added, making the task increasingly difficult. |
| **MULTIPLE OBJECT TRACKING (MOT) TASK – Visuo-cognitive Attention**  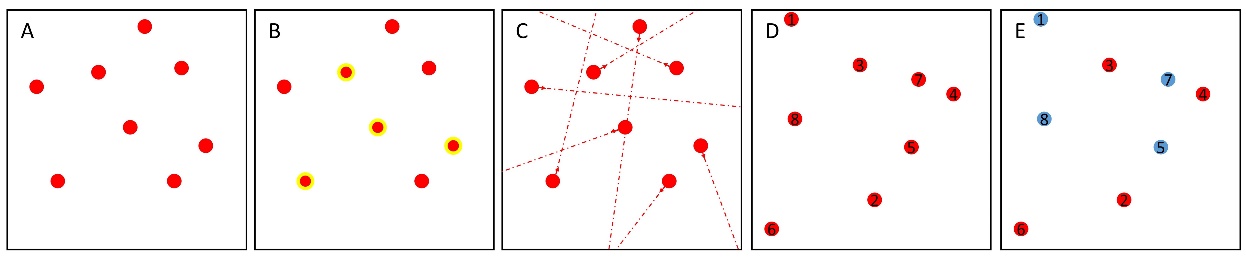  A) Eight identical spheres were displayed within a 3D Cube. B) Four of them (the targets participants had to track) were highlighted during two seconds. C) All spheres randomly moved inside the cube for eight seconds. D) Spheres were numerated and participants had to select the targets. E) A feedback was provided. |

Figure 1. Illustration and description of the visuo-attentional tasks.

| **FLANKER TASK – Visual Inhibition**  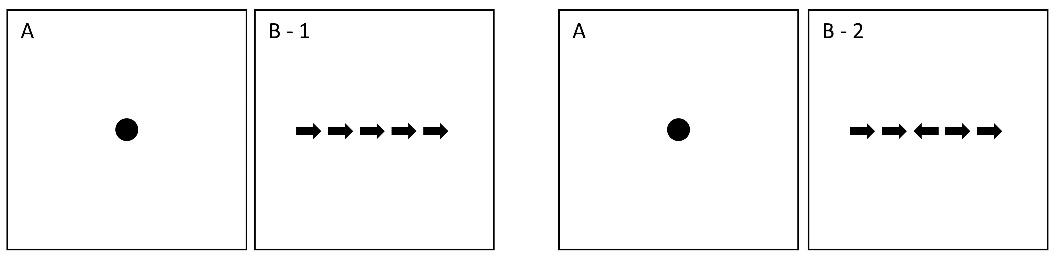  A) Participants first looked at the center of the screen and experience either congruent (B-1) or incongruent (B-2) trials. In all cases, they had to detect the direction of the central arrow as quickly as possible and push the appropriate button on the keyboard. |
| --- |
| **STOP-SIGNAL TASK (SST) – Response Inhibition**  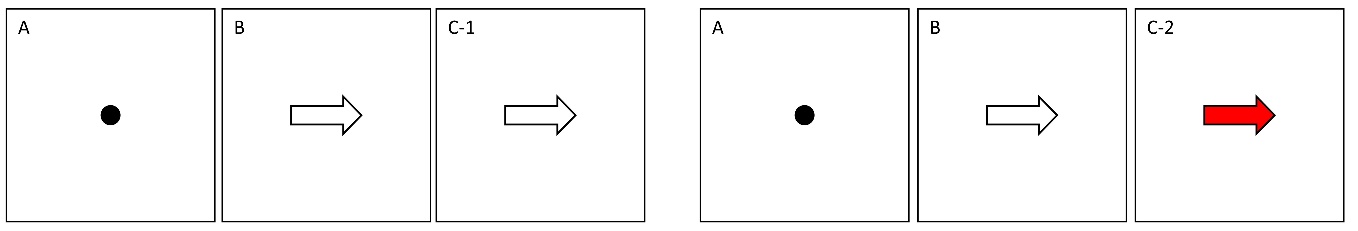  A) Participants first looked at the center of the screen. B) When one arrow appeared, participants must initiate the movement to give the orientation of the arrow. C-1) If the arrow remained white (standard trial), participants pushed the appropriate button on the keyboard. C-2) If the arrow turned red (stop trial), participants were asked not to respond. |
| **N-BACK TASK – Working Memory**  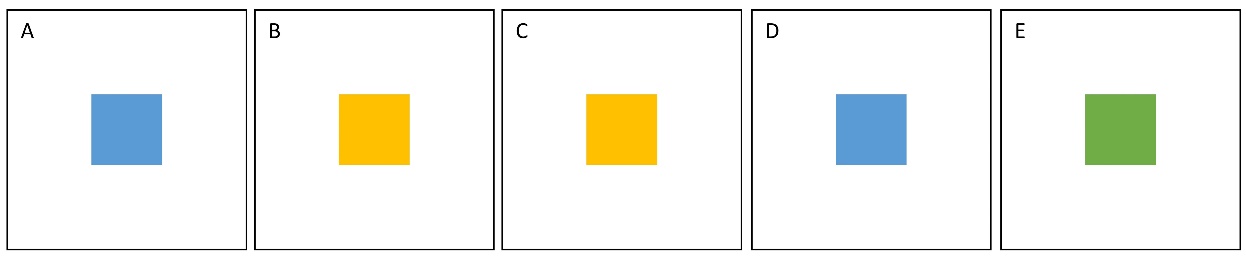  The images A to E represent a sequence of coloured squares displayed in the screen one at a time. In the 3-back task, participants had to respond at time D as the square displayed (D) was the same as the one presented 3 steps back (A). |
| **CORSI TASK – Spatial Working Memory**  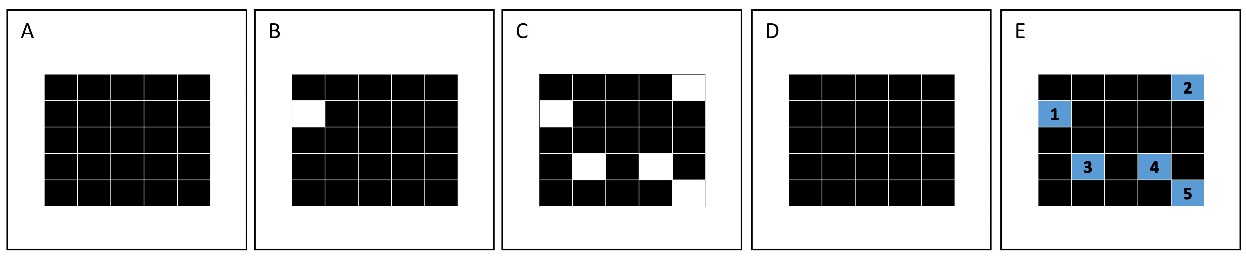  A) An empty grid was displayed on the screen. B) A first square appeared on the screen. C) All squares of the sequence were displayed one after the other. D) The grid returned to its initial state. E) Participants had to reproduce the sequence in its order of appearance. |
| **TRAIL MAKING TEST (TMT) – Cognitive Flexibility**  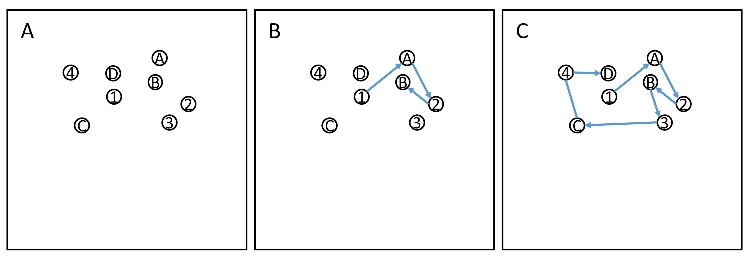  A) Participants discovered the numbers and letter of the test board. B and C) They had to alternate between numbers and letters and complete the test as quickly as possible. |
| **TOWER OF HANOI – Spatial Planification**  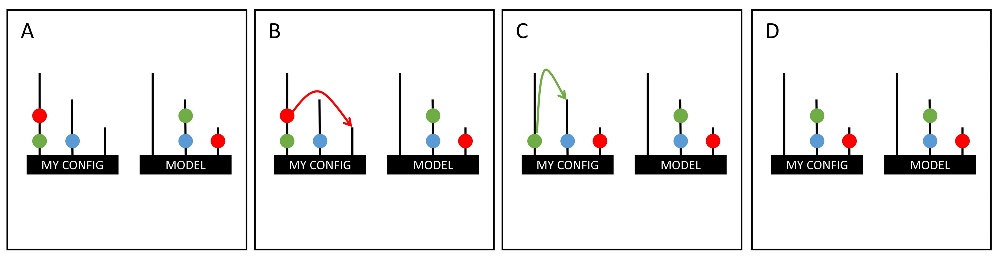  A) Participants discovered the model to be reproduced. B) Participants moved the red ball to the latter cylinder. C) Participants moved the green ball on the middle cylinder. D) The model was reproduced. |

Figure 2. Illustration and description of the cognitive tasks.


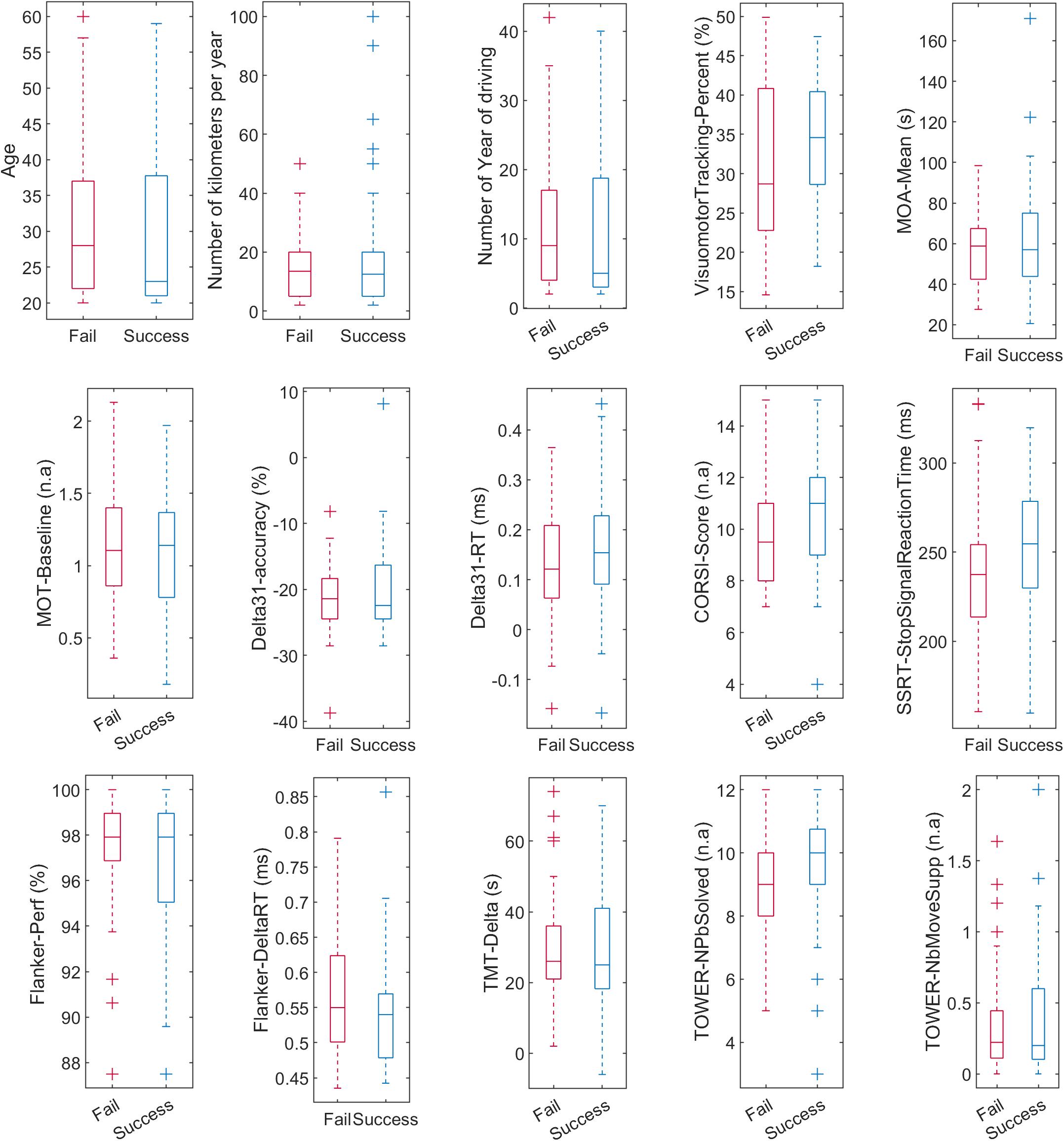


Figure 3. Box plot of average scores as a function of participant group (Fail, Success) for all individual abilities.


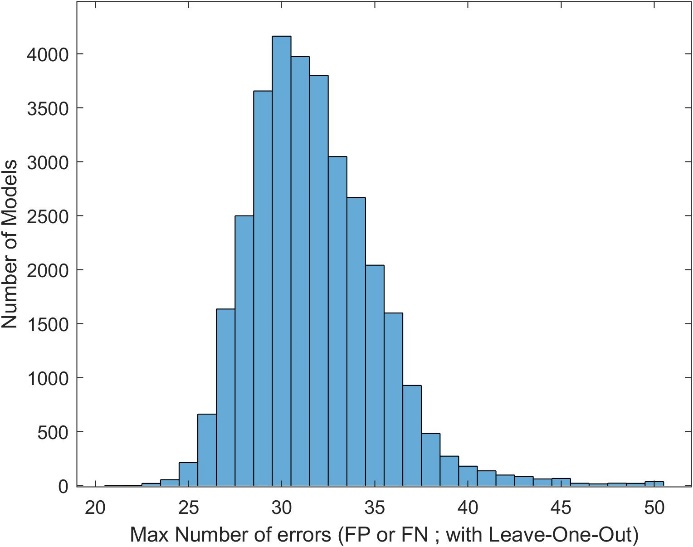


Figure 4. Classification performance of the 2¹⁵ – 1 models of PLS logistic regression in terms of the maximum number of errors of each model after leave-one-out procedure (x axis). Most of the models made between 25 and 40 errors. The worst model made 51 errors, while two models (discussed in the main body of the paper) minimised the number of errors to 21.

|  | Age |  |  |  |  |  |  |  |  |  |  |  |  |  |  |
| --- | --- | --- | --- | --- | --- | --- | --- | --- | --- | --- | --- | --- | --- | --- | --- |
| Age | X | **DrivingExp**  **Km** |  |  |  |  |  |  |  |  |  |  |  |  |  |
| **DrivingExp_Km** | **0,16** | **x** | DrivingExp  Years |  |  |  |  |  |  |  |  |  |  |  |  |
| DrivingExp_Years | 0,90** | **0,20*** | x | **VMC**  **Percent** |  |  |  |  |  |  |  |  |  |  |  |
| **VMC_Percent** | **-0,41**** | **0,08** | **-0,39**** | **x** | MOA  Time |  |  |  |  |  |  |  |  |  |  |
| MOA_Time | -0,25* | **0,16** | -0,19 | **0,36**** | x | MOT  TrackingSpeed |  |  |  |  |  |  |  |  |  |
| MOT_TrackingSpeed | -0,32** | **-0,10** | -0,39** | **0,22*** | 0,25* | x | Nback  Acc |  |  |  |  |  |  |  |  |
| Nback_Acc | -0,24* | **-0,18** | -0,15 | **0,14** | 0,07 | -0,05 | x | Nback  RT |  |  |  |  |  |  |  |
| Nback_RT | 0,08 | **0,08** | 0,09 | **-0,17** | -0,16 | -0,03 | 0,08 | x | **CORSI**  **Score** |  |  |  |  |  |  |
| **CORSI_Score** | **-0,26*** | **-0,03** | **-0,21*** | **0,19** | **0,19** | **0,45**** | **0,06** | **-0,14** | **x** | **SSRT** |  |  |  |  |  |
| **SSRT** | **-0,17** | **-0,09** | **-0,09** | **0,15** | **0,32**** | **0,24*** | **0,01** | **-0,11** | **-0,01** | **x** | **Flanker**  **Acc** |  |  |  |  |
| **Flanker_Acc** | **0,07** | **0,02** | **0,07** | **-0,10** | **-0,06** | **0,00** | **0,07** | **0,19** | **0,05** | **0,07** | **x** | Flanker  RT |  |  |  |
| Flanker_RT | -0,52** | **-0,09** | -0,55** | **0,49**** | 0,36** | 0,47** | 0,15 | -0,25* | **0,34**** | **0,18** | **-0,15** | x | TMT |  |  |
| TMT | -0,09 | **-0,12** | -0,06 | **0,19** | 0,16 | 0,39** | 0,07 | -0,32** | **0,28**** | **0,13** | **-0,08** | 0,37** | x | Tower  Acc |  |
| Tower_Acc | -0,31** | **-0,01** | -0,29** | **0,27**** | 0,25* | 0,24* | 0,15 | -0,21* | **0,23*** | **0,12** | **-0,08** | 0,39** | 0,20* | x | Tower  Supp |
| Tower_Supp | -0,15 | **-0,14** | -0,12 | **0,15** | 0,10 | 0,17 | -0,04 | -0,03 | **0,08** | **0,13** | **-0,24*** | 0,11 | -0,05 | 0,22* | x |
| Takeover Performance  (Y) | -0.08 | **0.10** | -0.04 | **0.17** | 0.08 | 0.03 | 0.12 | -0.11 | **0.21*** | **-0.19*** | **-0.16** | 0.15 | 0.01 | 0.06 | 0.03 |

Table 1. Table of correlation between the fifteen variables considered in the study. The five variables retained in the final model have been highlighted in bold. * p < 0.05; ** p < 0.01

|  | **Mean Fail** | **Mean Success** | **t-value** | **p-value** |
| --- | --- | --- | --- | --- |
| **Age** | 31,10 | 29,43 | -0,80 | 0,42 |
| **DrivingExp_Km** | 14846,05 | 18340,00 | 1,05 | 0,29 |
| **DrivingExp_Years** | 11,80 | 11,05 | -0,39 | 0,70 |
| **VMC_Percent** | 31,61 | 34,62 | 1,81 | 0,07 |
| **MOA_Time** | 58,14 | 61,69 | 0,84 | 0,40 |
| **MOT_TrackingSpeed** | 1,14 | 1,12 | -0,29 | 0,76 |
| **Nback_Acc** | -21,27 | -19,83 | 1,26 | 0,21 |
| **Nback_RT** | 0,13 | 0,16 | 1,20 | 0,23 |
| **CORSI_Score** | 9,56 | 10,40 | 2,25 | **0,03*** |
| **SSRT** | 236,58 | 252,07 | 2,11 | **0,04*** |
| **Flanker_Acc** | 0,98 | 0,96 | -1,73 | 0,09 |
| **Flanker_RT** | 0,57 | 0,54 | -1,69 | 0,10 |
| **TMT** | 30,50 | 30,06 | -0,14 | 0,88 |
| **Tower_Acc** | 9,18 | 9,36 | 0,61 | 0,54 |
| **Tower_Supp** | 0,35 | 0,37 | 0,35 | 0,73 |
|  |  |  |  |  |
| **SA Score** | 1,16 | 1,47 | 2,10 | **0,04*** |

Table 2. Table of two sample t-tests for the fifteen variables considered, as well as situation awareness score. *p < 0.05
